# Mitochondrial phylogeography of grassland caterpillars (Lepidoptera: Lymantriinae: *Gynaephora*) endemic to the Qinghai–Tibetan plateau

**DOI:** 10.1002/ece3.70270

**Published:** 2024-09-15

**Authors:** Ming‐Long Yuan, Ming‐Hui Bao, Qi‐Lin Zhang, Zhong‐Long Guo, Min Li, Juan Wang

**Affiliations:** ^1^ State Key Laboratory of Herbage Improvement and Grassland Agro‐Ecosystems, College of Pastoral Agriculture Science and Technology Lanzhou University Lanzhou China; ^2^ Faculty of Life Science and Technology Kunming University of Science and Technology Kunming China; ^3^ Co‐Innovation Center for Sustainable Forestry in Southern China, College of Biology and the Environment Nanjing Forestry University Nanjing China

**Keywords:** biogeography, divergence time estimation, gene flow, insects, moths, population genetic structure, Qinghai–Tibetan Plateau, speciation

## Abstract

Grassland caterpillars (Lepidoptera: Lymantriinae: *Gynaephora*) are the most damaging pests to alpine meadows in the Qinghai–Tibetan Plateau (QTP). Here, we conducted extensive sampling from 39 geographic populations covering almost the entire distribution of the eight QTP *Gynaephora* (Hübner) species to investigate phylogeographic patterns and speciation based on two mitochondrial genes (*COI* and *ND5*). A total of 40 haplotypes were detected in the 39 populations, with >70% of all haplotypes not shared between populations. The monophyletic QTP *Gynaephora* migrated from non‐QTP regions during the Pliocene, corresponding to the uplift of the QTP, suggesting a mode of transport into the QTP. Among the eight QTP *Gynaephora* species described by morphological characteristics, two species (*G. alpherakii* and *G. menyuanensis*) were recovered as monophyletic groups (Clades B and C), while the remaining six formed two monophyletic clades: Clade A (*G. qinghaiensis*, *G. jiuzhiensis*, and *G. qumalaiensis*) and Clade D (*G. aureata*, *G. ruoergensis*, and *G. minora*). These results suggested that the number of the QTP *Gynaephora* species may be overestimated and further studies based on both morphological and nuclear gene data are needed. Genetic differentiation and speciation of the QTP *Gynaephora* were likely driven by the QTP uplifts and associated climate fluctuations during the Pleistocene, indicated by divergence time estimation, suggesting that isolation and subsequent divergence was the dominant mode of speciation. The Sanjiangyuan region (i.e., Clade A, characterized by high genetic diversity) may have been a glacial refugium of the QTP *Gynaephora*, as supported by analyses of gene flow and biogeography. High levels of genetic diversity were found in QTP *Gynaephora*, without population expansion, which may explain the high‐altitude adaptation and outbreaks of grassland caterpillars in alpine meadows of the QTP. This study provides the largest phylogeographic analysis of QTP *Gynaephora* and improves our understanding of the diversity and speciation of QTP insects.

## Graphical abstract

We conducted extensive sampling from 39 geographic populations covering almost the entire distribution of the eight Gynaephora species in the QTP to investigate phylogeographic patterns and speciation based on two mitochondrial genes. The results suggested that the number of the QTP Gynaephora species may be overestimated and further studies based on both morphological and nuclear gene data are needed. Genetic differentiation and speciation were driven by intensive uplifts of the QTP and associated climate fluctuations during the Pleistocene, suggesting that isolation and subsequent divergence was the dominant mode of speciation.

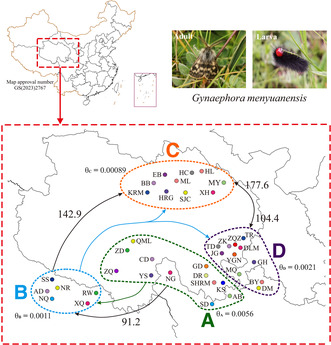

## 1 INTRODUCTION

The Qinghai–Tibetan Plateau (QTP) has the highest average altitude in the world and is characterized by extreme environmental features (e.g., hypoxia, cold climate, and high levels of ultraviolet radiation). Extensive uplifts of the QTP since the Miocene period (~23 million years ago, Ma) have caused dramatic climatic and environmental shifts (Harrison et al., 1992; Jia et al., 2003; Wu et al., 2001). Both geological and climatic changes are well‐established drivers of species diversification and speciation patterns in various QTP plants and animals (Favre et al., 2015; Lei et al., 2014; Xing & Ree, 2017). Thus, the QTP and adjacent regions have numerous endemic species and have been recognized as biodiversity hotspots (Myers et al., 2000).

During the past few decades, the QTP has been considered a “natural laboratory” for exploring adaptation and evolution of plants and animals (Jiang et al., 2018). Phylogeographic analyses of many QTP taxa, including mammals (Liu et al., 2012; Ruan et al., 2005), birds (Zhang & Fritsch, 2010), amphibians (Che et al., 2010; Yan et al., 2013; Zhou et al., 2012), and fishes (Chen et al., 2004; Peng et al., 2006; Qi et al., 2006), have been reported. An increasing number of recent studies have focused on the differentiation and speciation of insects inhabiting the QTP, e.g., *Gnaptorina* (Coleoptera: Tenebrionidae) (Li et al., 2021) and *Pseudabris hingstoni* (Coleoptera: Meloidae) (Wang, 2021). These previous studies have reported associations between speciation patterns and the QTP uplifts (as well as associated climatic changes) and highlight the diversity in phylogeographic structures and divergence processes among taxa. For example, some species used southeastern regions of the QTP as a large refugium during the major glaciations in the Pleistocene (Fan et al., 2012; Liu et al., 2015; Wang et al., 2017), while glacial refugia were maintained in the central platform of the QTP for other native species (Liu et al., 2013; Muellner‐Riehl, 2019; Tang et al., 2010).

Grassland caterpillars (Lepidoptera: Erebidae: Lymantriinae: *Gynaephora*) are among the most damaging insect pests to alpine meadows of the QTP (Yan, 2006; Yuan et al., 2015; Zhang & Yuan, 2013). *Gynaephora* (Hübner) is a small genus, with only one species described in China before the 1970s and eight species described to date according to morphological characteristics (Yan, 2006; Zhang & Yuan, 2013). The number of QTP *Gynaephora* accounted for over half of the 15 known *Gynaephora* species worldwide, making the QTP the speciation center of *Gynaephora* (Yan, 2006; Zhang & Yuan, 2013). It is noteworthy that the QTP *Gynaephora* species show high similarity in morphological characteristics (e.g., overall size, wing color, and external genitalia shape) and most species are locally distributed, e.g., *G. ruoergensis* and *G. minora* are endemic to Ruoergai County of Sichuan Province (Chou & Ying, 1979; Yan, 2006; Zhang & Yuan, 2013). To assess the taxonomic status of the eight QTP *Gynaephora* species, our previous study firstly performed molecular phylogenetic and species delimitation analyses for all QTP *Gynaephora* species collected from 15 geographic populations by using two mitochondrial genes (*COI* and *ND5*) and two nuclear genes (*EF‐1α* and *GAPDH*) (Yuan et al., 2015). Our results did not recover each of all eight *Gynaephora* species, reducing the number of QTP *Gynaephora* species to six, i.e., individuals of three *Gynaephora* species (*G*. *aureata*, *G*. *ruoergensis*, and *G*. *minora*) showed admixture and were characterized by very low genetic distances (Yuan et al., 2015). The three *Gynaephora* species were also supported as a single species based on mitogenomic data (Yuan et al., 2018). Therefore, sampling of more geographic populations and individuals is necessary to explore species statuses, speciation, and biogeographic patterns of QTP *Gynaephora*.

Here, we conducted extensive sampling from 39 geographic populations in alpine meadows, covering the majority of the distribution of each of the eight QTP *Gynaephora* species to investigate phylogeography and speciation based on two mitochondrial genes (*COI* and *ND5*). We aimed to address the following three questions. (1) How many *Gynaephora* species are in the QTP? (2) Do QTP *Gynaephora* species have high genetic diversity? (3) What are the speciation patterns in QTP *Gynaephora*. This study provides the largest phylogeographic analysis of QTP *Gynaephora* and expands our general understanding of the diversity and speciation of the QTP insects.

## 2 MATERIALS AND METHODS

### 2.1 Sampling and DNA extraction

**FIGURE 1:**
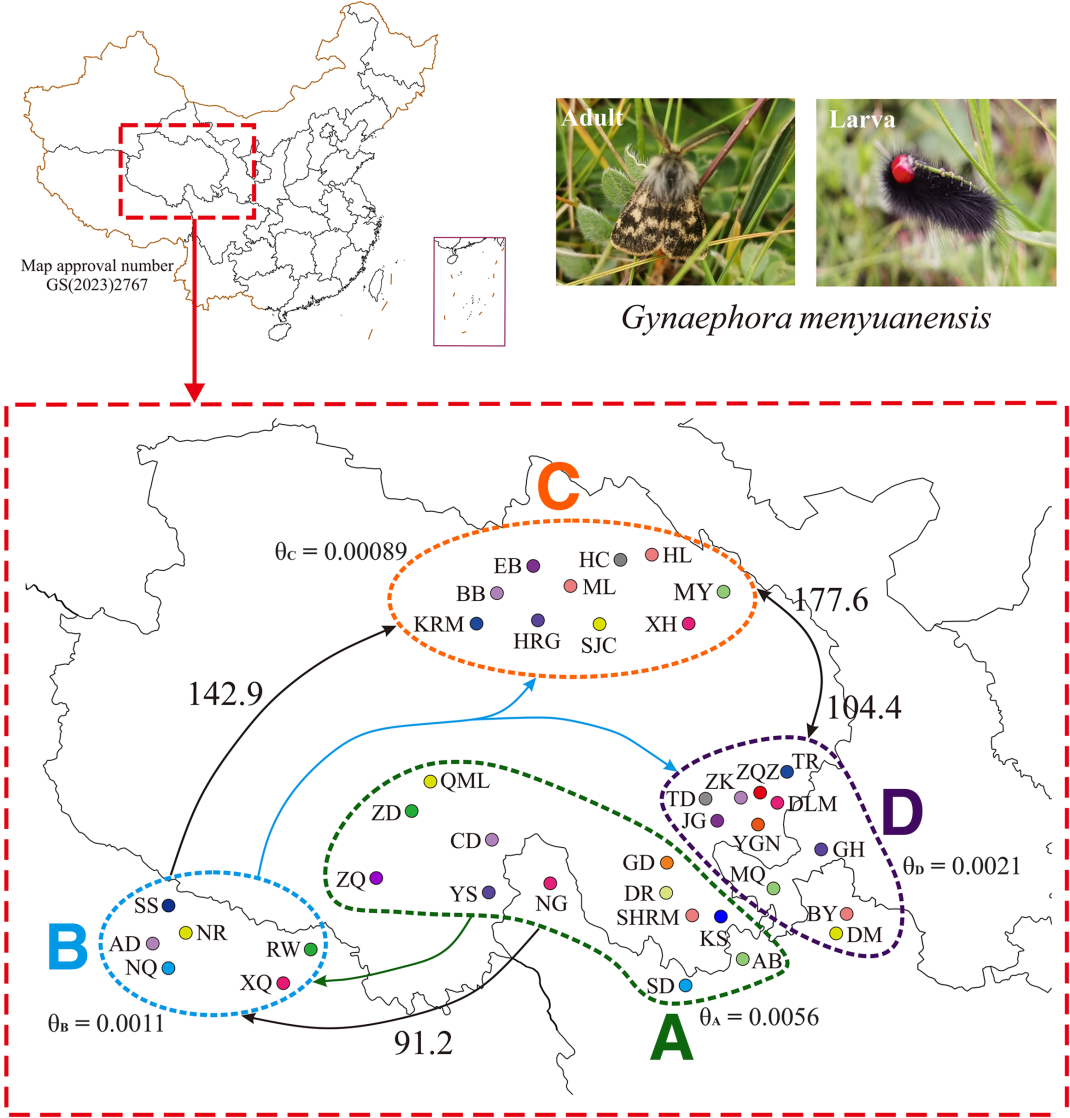
Sampling sites of 39 geographical populations and population structure of the QTP *Gynaephora* species based on the two mitochondrial genes (*COI* and *ND5*). Four groups are indicated by A–D, corresponding to four clades in Figures 2 and 3. See Table 1 for full names of the 39 geographical populations. Colored arrows indicate the dispersal routes inferred by a biogeographic analysis using RASP. Black arrows indicate the direction of gene flow and migration rates are provided above the lines. Effective population sizes (θ) are shown for Clades A–D.

A total of 488 individual specimens from 39 geographical localities were used in this study, out of those 145 individuals from 15 populations were retrieved from our previous study (Yuan et al., 2015). Our sampling included all the eight *Gynaephora* species inhabiting the QTP and covered most of the distribution of grassland caterpillars in the QTP meadows. Detailed sampling information is provided in Figure 1 and Table 1. All *Gynaephora* specimens were hand‐netted in alpine meadows of the QTP during July–August between 2013 and 2016. All specimens were stored in absolute ethyl alcohol in the field and transferred to −20°C until they were used for DNA extraction. Total genomic DNA was extracted from each specimen using the DNA Extraction Kit (Tiangen, Beijing, China) following the manufacturer's protocol.

**TABLE 1:** Sampling information and haplotype distributions for 39 geographic populations of the eight QTP *Gynaephora* species.

Species	Population ID	Locality	Sample size	Haplotype	Clade in Figures 1, 2, 3	Longitude	Latitude	Altitude (m)
*G. jiuzhiensis*	AB	Aba County, Sichuan Province, China	10	H1, H2	Clade A	101°45′ E	32°54′ N	3350
*G. jiuzhiensis*	DR	Dari County, Qinghai Province, China	9	H14, H15	Clade A	100°03′ E	33°28′ N	4150
*G. jiuzhiensis*	GD	Gande County, Qinghai Province, China	9	H16	Clade A	100°14′ E	34°13′ N	4100
*G. jiuzhiensis*	KS	Jiuzhi County, Qinghai Province, China	13	H2	Clade A	101°29′ E	33°22′ N	3650
*G. jiuzhiensis*	SHRM	Jiuzhi County, Qinghai Province, China	10	H2	Clade A	101°19′ E	33°22′ N	4050
*G. qinghaiensis*	CD	Chenduo County, Qinghai Province, China	10	H10	Clade A	97°34′ E	33°34′ N	4000
*G. qinghaiensis*	NG	Shiqua County, Sichuan Province, China	20	H23, H24, H25, H26	Clade A	98°12′ E	32°96′ N	4400
*G. qinghaiensis*	SD	Seda County, Sichuan Province, China	20	H33	Clade A	100°33′ E	32°27′ N	4000
*G. qinghaiensis*	YS	Yushu County, Qinghai Province, China	10	H38	Clade A	97°13′ E	32°83′ N	4000
*G. qumalaiensis*	QML	Qumalai County, Qinghai Province, China	9	H30, H31, H32	Clade A	95°79′ E	34°07′ N	4550
*G. qumalaiensis*	ZD	Zhiduo County, Qinghai Province, China	10	H30, H39	Clade A	95°46′ E	33°79′ N	4500
*G. qumalaiensis*	ZQ	Zaduo County, Qinghai Province, China	8	H30	Clade A	95°21′ E	33°22′ N	4250
*G. alpherakii*	AD	Anduo County, the Tibet Autonomous Region, China	20	H3	Clade B	91°68′ E	32°03′ N	4900
*G. alpherakii*	NQ	Naqu County, the Tibet Autonomous Region, China	10	H3	Clade B	92°04′ E	31°48′ N	4550
*G. alpherakii*	NR	Nierong County, the Tibet Autonomous Region, China	16	H3, H27, H28, H29	Clade B	92°26′ E	31°74′ N	4700
*G. alpherakii*	RW	Suoxian County, the Tibet Autonomous Region, China	20	H3	Clade B	93°82′ E	31°72′ N	3600
*G. alpherakii*	SS	Anduo County, the Tibet Autonomous Region, China	18	H3	Clade B	92°05′ E	31°93′ N	4800
*G. alpherakii*	XQ	Biru County, the Tibet Autonomous Region, China	20	H3	Clade B	93°83′ E	31°79′ N	4400
*G. menyuanensis*	BB	Qilian County, Qinghai Province, China	10	H4, H5	Clade C	100°26′ E	38°16′ N	2800
*G. menyuanensis*	EB	Qilian County, Qinghai Province, China	20	H4	Clade C	100°94′ E	37°95′ N	3400
*G. menyuanensis*	HC	Qilian County, Qinghai Province, China	10	H5, H18	Clade C	101°19′ E	37°62′ N	3200
*G. menyuanensis*	HL	Qilian County, Qinghai Province, China	10	H5, H18	Clade C	100°78′ E	37°70′ N	3400
*G. menyuanensis*	HRG	Gangcha County, Qinghai Province, China	12	H4, H19	Clade C	100°37′ E	37°19′ N	3400
*G. menyuanensis*	KRM	Tianjun County, Qinghai Province, China	16	H4	Clade C	98°84′ E	37°33′ N	3200
*G. menyuanensis*	ML	Qilian County, Qinghai Province, China	10	H5	Clade C	100°44′ E	37°56′ N	3400
*G. menyuanensis*	MY	Menyuan County, Qinghai Province, China	10	H5	Clade C	101°18′ E	37°38′ N	3100
*G. menyuanensis*	SJC	Gangcha County, Qinghai Province, China	16	H4, H5, H34	Clade C	100°16′ E	37°35′ N	3400
*G. menyuanensis*	XH	Haiyan County, Qinghai Province, China	12	H4	Clade C	100°91′ E	37°03′ N	3050
*G. aureata*	DLM	Zeku County, Qinghai Province, China	11	H11	Clade D	101°55′ E	35°02′ N	3600
*G. aureata*	GH	Luqu County, Gansu Province, China	15	H7, H17	Clade D	102°36′ E	34°24′ N	3450
*G. aureata*	JG	Maqin County, Qinghai Province, China	10	H20, H21	Clade D	100°42′ E	34°39′ N	3200
*G. aureata*	MQ	Maqu County, Gansu Province, China	10	H22	Clade D	101°52′ E	33°50′ N	3600
*G. aureata*	TD	Tongde County, Qinghai Province, China	10	H35	Clade D	100°48′ E	34°42′ N	3400
*G. aureata*	TR	Tongren County, Qinghai Province, China	10	H7, H11	Clade D	101°47′ E	35°12′ N	3500
*G. aureata*	YGN	Henan County, Qinghai Province, China	13	H11, H36, H37	Clade D	101°36′ E	34°46′ N	3700
*G. aureata*	ZK	Zeku County, Qinghai Province, China	10	H11, H40	Clade D	101°31′ E	34°54′ N	3650
*G. aureata*	ZQZ	Zeku County, Qinghai Province, China	11	H7, H11	Clade D	101°45′ E	35°00′ N	3650
*G. minora*	DM	Ruoergai County, Sichuan Province, China	10	H6, H7, H8, H9, H12, H13	Clade D	102°57′ E	33°18′ N	3450
*G. ruoergensis*	BY	Ruoergai County, Sichuan Province, China	10	H6, H7, H8, H9	Clade D	103°06′ E	33°34′ N	3450

### 2.2 PCR amplification and sequencing

Two mitochondrial genes (*COI* and *ND5*) were amplified using the same primers and procedures described in our previous study (Yuan et al., 2015). All PCR products were purified using a DNA Gel Purification Kit (Omega, Norwalk, CT, USA) and sequenced in both directions by Sanger sequencing using the same PCR primers.

Sequences of the two mitochondrial genes (*COI* and *ND5*) were initially aligned using ClustalW in MEGA 5.10 (Tamura et al., 2011) with default parameters to check for stop codons or indels, which could reveal mitochondrial pseudogenes. All sequences newly obtained in this study have been deposited in GenBank under accession numbers OP574353–OP574695 and OP577499–OP577841. Then, sequences of *COI* and *ND5* were concatenated using DAMBE 7.2.6 (Xia, 2018). Mitochondrial haplotypes were identified using DNASP 5.10 (Librado & Rozas, 2009).

### 2.3 Phylogenetic analysis

A total of 11 *Gynaephora* species were included in phylogenetic analyses, i.e. eight QTP *Gynaephora* species, *G. groenlandica*, *G. rossii*, and *G. selenitica*. We selected *Lymantria dispar* (NC_012893) as the outgroup, according to our previous study (Yuan et al., 2015). For each gene, substitution saturation was evaluated using DAMBE 7.2.6 (Xia, 2018). A lack of evidence for saturation indicated that all codon positions can be used for the phylogenetic analysis. The partitioning schemes and corresponding nucleotide substitution models for the dataset (*COI* + *ND5*) were selected by PartitionFinder 1.1.1 (Lanfear et al., 2012), partitioned by genes and codon positions as in our previous study (Yuan et al., 2015). Single partitioning scheme with the HKY + I + G model was used for the following phylogenetic analyses. The phylogenetic tree was constructed based on the mitochondrial dataset (*COI* + *ND5*) using maximum likelihood (ML) and Bayesian inference (BI). Both BI and ML analyses were conducted using the CIPRES Science Gateway 3.3 (Miller et al., 2010). The ML analysis was carried out using RAxML‐HPC2 on XSEDE 8.0.24 (Stamatakis, 2014) with the GTRGAMMA model, and 1000 bootstrap (BS) replicates were used to estimate node reliability. The BI analysis was conducted using MrBayes 3.2.2 (Ronquist et al., 2012) on XSEDE. Four Markov chains (three hot and one cold chain) were independently run two times for 1 × 10^6^ generations, with sampling every 1000 generations. Chain congruence was assessed by the effective sample size (ESS) (>100) and the potential scale reduction factor (PSRF) (approximately 1.0), as recommended in MrBayes 3.2.2 documentation (Ronquist et al., 2012). The first 25% of samples were discarded as burn‐in, and the remaining samples were used to construct a 50% majority‐rule consensus tree. Bayesian posterior probabilities (PP) were calculated. Furthermore, a median‐joining network was generated for all mitochondrial haplotypes using Network 4.6.0.0 (Bandelt et al., 1999).

### 2.4 Genetic diversity and differentiation

Haplotype diversity (*h*) and nucleotide diversity (*π*) for all 39 geographic populations and each of the four clades (based on the population structure analysis) were calculated using DnaSP 5.10 (Librado & Rozas, 2009). Genetic differentiation (*F*_ST_) between the 39 geographic populations was calculated using Arlequin 3.5 (Excoffier & Lischer, 2010). The significance of *F*_ST_ between populations was tested by 10,000 permutations in Arlequin 3.5 (Excoffier & Lischer, 2010). To investigate genetic structuring between sampling locations, an analysis of molecular variance (AMOVA) (Excoffier et al., 1992) was performed with Arlequin 3.5 (Excoffier & Lischer, 2010). Mantel tests were performed using Isolation By Distance Web Service (IBDWS) (Jensen et al., 2005) to test the correlation between genetic distance and geographic distance.

### 2.5 Population demography

The historical population demographics of QTP *Gynaephora* species was investigated by neutrality tests (Fu's *Fs*, Fu and Li's F* and D*, and Tajima's D) and pairwise mismatch distributions for all 39 sampling locations combined and each of four clades separately using DnaSP 5.10 (Librado & Rozas, 2009). Fu's *Fs*, Fu and Li's F* and D*, and Tajima's *D* were calculated to detected population demography (Fu, 1997; Tajima, 1989). Significant negative *F*_S_ values can be taken as evidence for expansions, while positive values might result from population subdivision or a recent population bottleneck (Fu, 1997). For the mismatch distribution analysis, a unimodal distribution indicates a recent demographic expansion, while multimodal distributions indicate population stability (Harpending et al., 1998; Rogers & Harpending, 1992).

### 2.6 Divergence time estimation

Divergence times of the QTP *Gynaephora* were estimated by a molecular clock approach implemented in BEAST 1.8.1 (Drummond et al., 2012) using the phylogenetic tree of haplotypes as a constraint tree. A lognormal prior was used for *COI* and the mean substitution rate was set to 0.0115 per site per million years (Ma). We scaled the *ND5* substitution rate to the mean *COI* rate (according to the K2P genetic distances between *COI* and *ND5*), following the method described in our previous study (Yuan et al., 2018). The following settings were used: linked tree model, HKY + I substitution model for each mitochondrial gene, strict clock, Yule model of speciation, and default values for the remaining parameters. Chain convergence was checked using the ESS implemented in Tracer 1.5 (Rambaut et al., 2018). Posterior estimates of *Gynaephora* ages and 95% highest posterior densities (HPD) were summarized using TreeAnnotator v1.8.1 (Drummond et al., 2012). All BEAST analyses were conducted on the CIPRES Science Gateway 3.3 (Miller et al., 2010).

### 2.7 Biogeographical analysis

Ancestral area reconstruction was conducted to investigate the biogeographical history of *Gynaephora* by using a Bayesian binary MCMC (BBM) method, as implemented in RASP 3.1 (Yu et al., 2015). Seven biogeographical areas were defined based on the species distributions and results of the phylogenetic analysis (Figure 1): the distributions of (a) *G. groenlandica*; (b) *G. rossii*; (c) *G. selenitica*; and (d–g) the four clades A–D in Figure 1. The BBM analysis was run with the following settings: 10.001 credible trees generating from the BEAST analysis were used, the maximum number of areas for all nodes was set to seven, and the remaining parameters were set to default values.

### 2.8 Modeling the gene flow

Gene flow between the four clades (A–D in Figure 1) identified by phylogenetic analyses was modeled by using a coalescent‐based approach implemented in Migrate‐n (Beerli, 2006; Beerli & Palczewski, 2010). This software estimates long‐term average values of gene flow and can be used to test predictions about refugial structure and the direction of migration from putative refugia. The Sanjiangyuan region, locating in the central and eastern QTP, might represent a refugium of the QTP *Gynaephora* during glacial periods, as has been inferred in previous studies of other taxa (Qiu et al., 2011; Yang et al., 2009). Therefore, to reduce the number of models, we first divided the 39 populations into two groups based on the phylogenetic results, i.e., group A (Clade A in Figure 1) and group BCD (Clades B–D in Figure 1), and constructed three migration models (M1–M3 Figure S2). Then, we tested 14 migration models for three groups (Clade A, Clade B, and Clades C + D) and nine migration models for four groups (Clades A–D in Figure 1; Figure S2).

Migrate‐n was used to estimate the migration rates (M) and effective population size (*θ*) parameters as well as the marginal likelihood of each gene flow model (Beerli, 2006). The gene flow models were ranked according to log Bayes factors (LBFs) calculated from the Bezier corrected marginal likelihoods of the data given the model. The marginal likelihoods for each model were approximated by thermodynamic integration of the MCMC over four heated chains (Metropolis coupled MCMC), as described previously (Beerli & Palczewski, 2010). We used a uniform prior for *θ* between 0 and 0.1 and a sampling window of 0.01 where we generate new proposals; for M, we used a uniform prior between 0 and 1000, with a window of 100 model. A four‐chain static heating scheme was used, and 100,000 trees were discarded as burn‐in before recording 1,000,000 steps with an increment of 100, resulting in 40 million samples. To evaluate convergence, we evaluated ESS (>10,000) and the posterior distributions of the parameters to determine whether they were unimodal smooth curves. Bayes factors (BFs) were calculated as a ratio of the marginal likelihoods to calculate model probabilities.

## 3 RESULTS

### 3.1 Population structure

**FIGURE 2:**
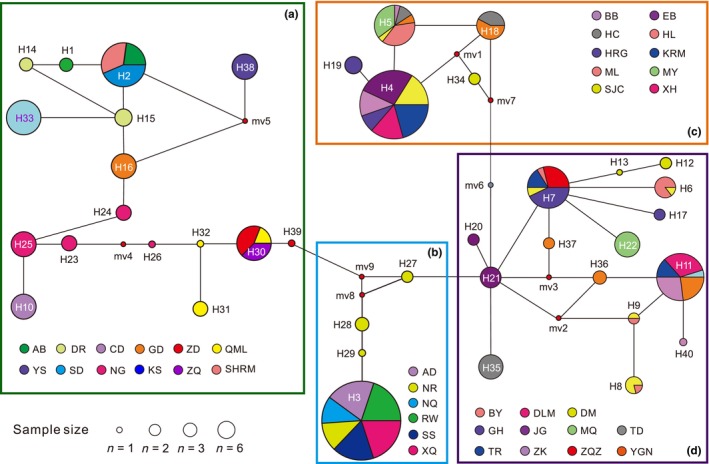
Median‐joining network of 40 haplotypes of QTP *Gynaephora* species. Four groups are indicated by (a)–(b), corresponding to four clades in Figures 1 and 3. See Table 1 for full names of the 39 geographical populations. The size of each circle (pie chart) indicates the number of individuals sharing this haplotype. The nine smallest circles (mv1–mv9) indicate hypothesized haplotypes.

A total of 40 haplotypes were detected for the combined mitochondrial gene dataset (*COI* and *ND5*) (Table 1). Among these haplotypes, each of 29 haplotypes was found only in one population, and the remaining 11 haplotypes were shared by multiple geographic populations (Table 1; Figure 2). Haplotypes H3 and H4 showed the highest frequencies, followed by H2, H5, H7, and H11 (Figure 2). Only one haplotype was detected in all 18 populations, and the DM population (six haplotypes) had the largest number of haplotypes (Table 1).

**FIGURE 3:**
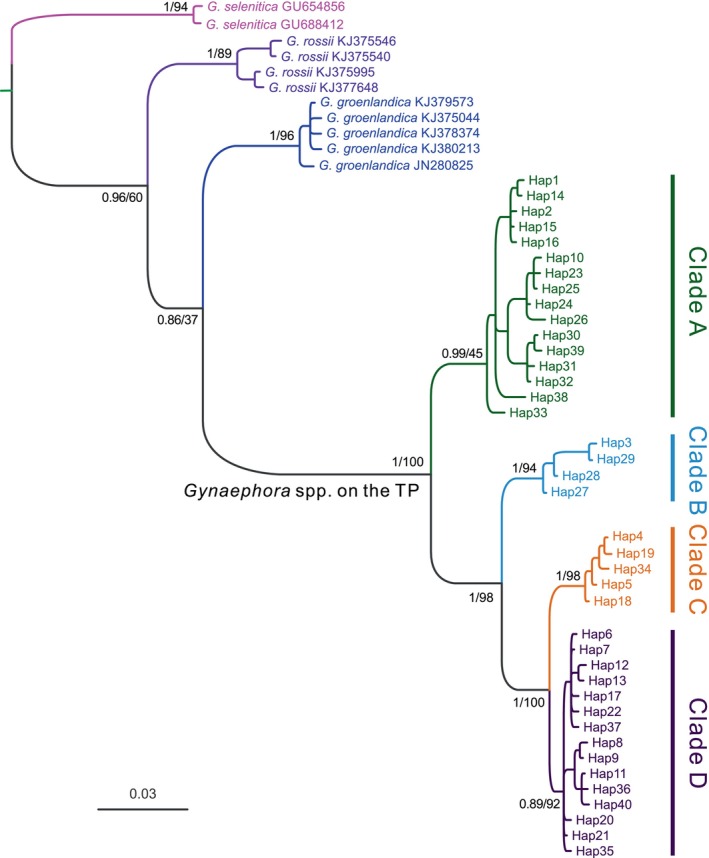
Bayesian phylogenetic trees of *Gynaephora* species based on two mitochondrial genes (*COI* and *ND5*). Posterior probabilities (left) and bootstrap support (right) values are shown above the main nodes.

Phylogenetic analyses based on 40 haplotypes using two methods (BI and ML) yielded nearly identical topologies (Figure 3). The QTP *Gynaephora* species formed a strongly supported monophyletic group (PP = 1.0, BS = 100) and consisted of four clades (A–D in Figures 1 and 3). Clade A (16 haplotypes in 12 populations) formed a sister group relationship to a clade containing the remaining three clades (Clades B–D), whereas Clade B (four haplotypes in six populations) was sister to Clade C (five haplotypes in 10 populations) and Clade D (15 haplotypes in 11 populations) (Figure 3). These four clades were also obtained by a haplotype network analysis (Figure 2).

### 3.2 Genetic diversity and differentiation

For QTP *Gynaephora* species overall, high levels of haplotype diversity (*h* = 0.913 ± 0.007) and nucleotide diversity (*π* = 2.288 ± 0.033) were observed (Table 2). Among the four clades (Figures 1, 2, 3), Clade A showed the highest genetic diversity (*H* = 16, *h* = 0.888 ± 0.012, and *π* = 0.641 ± 0.019%), followed by Clade D (*H* = 15, *h* = 0.837 ± 0.020, *π* = 0.201 ± 0.006%) and Clade C (*H* = 5, *h* = 0.567 ± 0.040, and *π* = 0.061 ± 0.006%), while Clade B (*H* = 4, *h* = 0.112 ± 0.042, and *π* = 0.073 ± 0.30%) showed the lowest genetic diversity (Table 2).

**TABLE 2:** Genetic diversity and neutrality tests for 39 geographic populations combined and for each of four clades of QTP *Gynaephora*.

	*n*	*H*	*h*	*π* (%)	Tajima's *D*	Fu and Li's D*	Fu and Li's F*	Fu's *F*_S_
All populations	488	40	0.913 ± 0.007	2.288 ± 0.033	2.98*	2.73*	3.42*	24.05**
Clade A	138	16	0.888 ± 0.012	0.641 ± 0.019	1.31^ns^	1.64*	1.81*	4.36*
Clade B	104	4	0.112 ± 0.042	0.073 ± 0.030	−1.45^ns^	1.42^ns^	0.47^ns^	1.25^ns^
Clade C	126	5	0.567 ± 0.040	0.061 ± 0.006	0.10^ns^	0.92^ns^	0.77^ns^	−0.06^ns^
Clade D	120	15	0.837 ± 0.020	0.201 ± 0.006	0.62^ns^	0.73^ns^	0.82^ns^	−2.57*

Abbreviations: *H*, number of haplotypes; *h*, haplotype diversity; *n*, sample size sequenced; π, nucleotide diversity.

*p < .05, **p < .001, ^ns^ *p* > .05. See Figures 1, 2, 3 for the four clades (Clades A–D).

Almost all pairwise *F*_ST_ values between the 39 sampling sites were significantly greater than 0 (Table S1), indicating substantial genetic differentiation between QTP *Gynaephora* populations. Mantel tests of the 39 QTP *Gynaephora* populations indicated that there was a significant positive correlation between genetic distances and geographic distances (*r* = .169–.517, *p* < .01; Table S2). Among the four clades, a significant correlation between genetic and geographic distances was found only for Clade A (*r* = .191–.457, *p* < .05; Table S2).

To further investigate genetic differentiation and genetic structuring between the 39 geographic populations we performed AMOVA for the four clades obtained from the phylogenetic and network analyses (Figures 1 and 2). A significantly positive *F*_CT_ (*F*_CT_ = 0.8701, *p* < .001; Table 3) suggested that geographic structuring existed among the examined populations. Genetic differentiation among the four clades accounted for 87.01% of the total genetic variance, whereas differentiation within clades only accounted for 1.35% of genetic variation, indicating a low gene flow among the four clades.

**TABLE 3:** Results of analysis of molecular variance (AMOVA) of four groups (i.e., Clades A–D in Figures 1, 2, 3).

Source of variation	df	Sum of squares	Variance components	Percentage of variation (%)	*F‐*statistic
Among groups	3	6019.55	16.23	87.01	*F*_CT_ = 0.8701*
Among populations within groups	35	947.68	2.17	11.64	*F*_SC_ = 0.8960*
Within populations	449	113.08	0.25	1.35	*F*_ST_ = 0.9865*

*p < .001.

### 3.3 Population demography

The mismatch distribution for the 40 haplotypes of the 39 geographic populations was multimodal (Figure S1), indicating that no obvious expansion occurred in the QTP *Gynaephora* species. Similar results were obtained for three clades (Clades A, B, and D), whereas Clade C showed a unimodal distribution, which could be due to either demographic expansion (population expansion) or to positive selection. In neutrality tests, significant negative values were not obtained for QTP *Gynaephora* as a whole or for Clades A, B, and C (Table 2). Significant positive values for each of four neutrality tests were found in all populations combined, whereas Fu's *F*_S_ value was significantly negative in Clade D (Table 2).

### 3.4 Divergence time estimation

**FIGURE 4:**
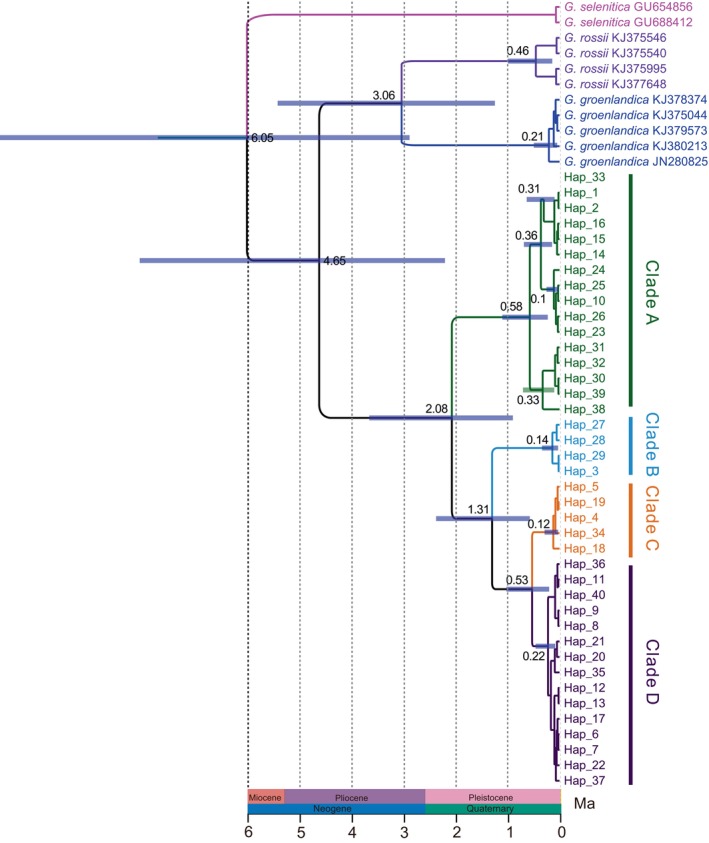
Divergence time estimation of the *Gynaephora* species using BEAST. Blue bars represent the 95% highest probability density interval. The numbers above nodes are the mean divergence times. Ma, million years ago.

Divergence time estimates for the genus *Gynaephora* based on a strict mitochondrial molecular clock are shown in Figure 4. Divergence within the genus was dated to the late Miocene, approximately 6.05 Ma (95% HPD: 2.9–10.83 Ma). The divergence of QTP *Gynaephora* species from non‐QTP species likely occurred during the early Pliocene, at around 4.65 Ma (95% HPD: 2.22–8.12 Ma). The earliest split within QTP *Gynaephora* was dated to the early Pleistocene at about 2.08 Ma (95% HPD: 0.90–3.68 Ma), followed by further splitting at around 1.31 Ma (95% HPD: 0.58–2.39 Ma). Clades C and D diverged about 0.53 Ma (95% HPD: 0.2–1.03 Ma).

### 3.5 Biogeographic reconstruction

**FIGURE 5:**
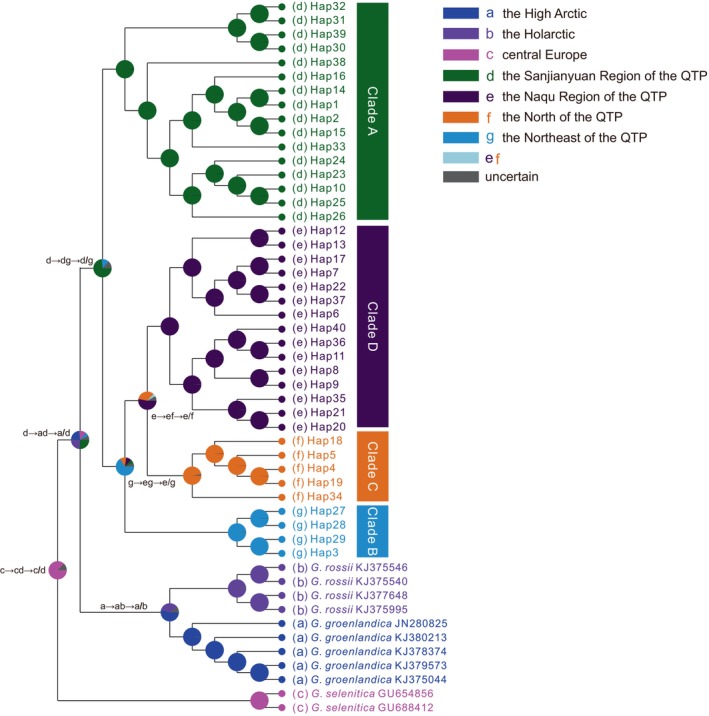
Biogeographic analysis of *Gynaephora* species using RASP. Clades A–D are the same as those in Figures 1, 2, 3. Present‐day ranges for each haplotype are color‐coded and drawn as small circles at the ends of terminal branches before haplotype names. Pie charts at internal nodes represent marginal probabilities for each alternative ancestral range (color codes).

The BBM biogeographic analysis revealed that the genus *Gynaephora* likely originated in central Europe (see Figure 5c for the distribution of the type species *G. selenitica*) with high probability; 12 dispersal and six vicariance events led to the colonization of current ranges with two expansion routes: northward dispersal to form several arctic *Gynaephora* species (e.g., *G. rossii* and *G. groenlandica*) and southward dispersal to form the QTP *Gynaephora* species (Figure 5). The common ancestor of the QTP *Gynaephora* species likely first dispersed to the Sanjiangyuan Region of the QTP (Clade A in Figures 1, 2, 3) and then to the Naqu Region, leading to the emergence of *G. alpherakii* by two westward dispersal events and one vicariance event, followed by dispersal to the North and Northeast QTP (Figure 5) and the split into two clades (Clades C and D in Figures 1, 2, 3).

### 3.6 Gene flow

A total of 26 models were evaluated using Migrate‐n (Table 4; Figure S2). The best‐supported models were M3 for two groups (A, BCD), M5 for three groups (A, B, CD), and M20 for four groups (Clades A–D) (Table 4; Figure S2). These best models consistently supported Clade A as the refugium with unidirectional gene flow from Clade A to Clade B (mean *Nm* = 91.2) and from Clade B to Clade C (mean *Nm* = 142.9) (Figure 1; Table S3). Bidirectional and asymmetrical gene flow were found between Clades C and D (Figure 1; Table S3). Theta estimates suggested that *Ne* was highest in Clade A and lowest in Clade C (Table S3).

**TABLE 4:** Results from model comparisons tested in Migrate‐n.

Model description	Model	Parameters	Bezier 1 mL	LBF	Probability
Two groups (A, BCD)					
Full model	M1	4 (2θ, 2M)	−5802.97	340.82	<0.01
BCD as refugium, 1 route	M2	3 (2θ, 1M)	−5646.05	26.98	<0.01
A as refugium, 1 route	M3	3 (2θ, 1M)	−5632.56	0	1
Three groups (A, B, CD)					
Full model	M4	9 (3θ, 6M)	−4968.95	456.0	<0.01
A as refugium, 1 route	M5	5 (3θ, 2M)	−4740.95	0	1
A as refugium, 2 routes	M6	6 (3θ, 3M)	−4811.54	141.18	<0.01
A as refugium, 2 routes	M7	6 (3θ, 3M)	−4872.75	263.6	<0.01
A as refugium, 2 routes	M8	7 (3θ, 4M)	−4838.25	194.6	<0.01
A as refugium, 1 route	M9	5 (3θ, 2M)	−4785.92	89.94	<0.01
A as refugium, 2 routes	M10	6 (3θ, 3M)	−4860.63	239.36	<0.01
A as refugium, 4 routes	M11	8 (3θ, 5M)	−4842.57	203.24	<0.01
A as refugium, 3 routes	M12	7 (3θ, 4M)	−4854.30	226.7	<0.01
A as refugium, 2 routes	M13	5 (3θ, 2M)	−4816.21	150.52	<0.01
A as refugium, 2 routes	M14	6 (3θ, 3M)	−4864.94	247.98	<0.01
A as refugium, 2 routes	M15	6 (3θ, 3M)	−4810.94	139.98	<0.01
A as refugium, 2 routes	M16	7 (3θ, 4M)	−4858.42	234.94	<0.01
A as refugium, 2 routes	M17	6 (3θ, 3M)	−4838.33	194.76	<0.01
Four groups (A, B, C, D)					
Full model, 4 populations	M18	16 (4θ, 12M)	−5463.89	1464.78	<0.01
A as refugium, 1 route	M19	7 (4θ, 3M)	−4744.14	25.28	<0.01
A as refugium, 2 routes	M20	8 (4θ, 4M)	−4731.50	0	1
A as refugium, 1 route	M21	7 (4θ, 3M)	−4782.24	101.48	<0.01
A as refugium, 2 routes	M22	8 (4θ, 4M)	−4842.89	222.78	<0.01
A as refugium, 2 routes	M23	7 (4θ, 3M)	−4800.06	137.12	<0.01
A as refugium, 3 routes	M24	8 (4θ, 4M)	−4864.18	265.36	<0.01
A as refugium, 2 routes	M25	8 (4θ, 4M)	−4933.58	404.16	<0.01
A as refugium, 3 routes	M26	9 (4θ, 5M)	−4805.27	147.54	<0.01

*Note*: Model descriptions are provided in Figure S2. The number of parameters for each model, Bezier approximation scores of log marginal likelihoods (1 mL), LBF, and model probabilities are shown. A–D correspond to Clades A–D in Figures 1, 2, 3.

## 4 DISCUSSION

### 4.1 The number of QTP *Gynaephora* species may be overestimated

The unique geographical and environmental conditions of the QTP led to a large number of endemic species. Eight *Gynaephora* species endemic to the QTP have been reported according to morphological characteristics (Yan, 2006; Zhang & Yuan, 2013), but were not well supported by molecular data in our previous studies (Yuan et al., 2015, 2018). In this study, by extensive sampling covering almost the whole geographical distribution and phylogenetic analyses, the number of valid species was further reduced to four monophyletic groups (Clades A–D in Figure 1). Clades B and C corresponded to *G. alpherakii* and *G. menyuanensis*, respectively. Clades A and D each contained multiple morphologically described species (Table 1). Among three species included in Clade A, *G. qinghaiensis* had a wide geographical distribution, while the two other *Gynaephora* (*G. qumalaiensis* and *G. jiuzhiensis*) are locally distributed (Yuan et al., 2015). *G. qinghaiensis* has long been considered the only species in alpine meadows of the QTP, i.e., grassland caterpillars destroying alpine meadows are all considered *G. qinghaiensis*. Although the Latin name *G. alpherakii* is frequently used to describe the QTP grassland caterpillars in the literature, Chou and Ying (1979) found that no specimens of grassland caterpillars sampled conformed to the morphological characteristics of *G. alpherakii*, questioning the existence of this species in the QTP. However, both our results and previous results consistently indicated that grassland caterpillars collected from multiple geographical localities in the Naqu Region of Tibet are an independent species, including those in Amdo County, the locality of the type specimen of *G. alpherakii*. Further studies including morphological characteristics are necessary to confirm whether this recovered monophyletic group is indeed *G. alpherakii*, as originally described.

Three *Gynaephora* species (*G. aureata*, *G. ruoergensis*, and *G. minora*) clustered together to form Clade D, as found in our previous study (Yuan et al., 2015). *G. aureata* is widely distributed, while the two other species are only distributed in Ruoergai County (Chou & Ying, 1979; Zhang & Yuan, 2013). The two sympatric species are morphologically similar, with obvious differences only in wing size (Chou & Ying, 1979), likely due to phenotypic plasticity. In addition, a sympatric distribution may facilitate relatively strong mitochondrial introgression due to interspecific hybridization. Among eight QTP *Gynaephora* species, *G. menyuanensis* is the most recently described and is mainly distributed in the northeastern QTP (Zhang & Yuan, 2013). This species can be effectively differentiated based on several morphological characteristics from other *Gynaephora* species. The validity of this species was also supported by molecular data in our present and previous study (Yuan et al., 2015), suggesting the independent species status of *G. menyuanensis*.

### 4.2 High genetic diversity in QTP *Gynaephora* species

Genetic diversity is a fundamental component of biodiversity. Generally, the higher the genetic diversity within a species, the greater the potential for adaptation in response to changing environments. Genetic diversity can be measured by two indices, i.e., haplotype diversity (*h*) and nucleotide diversity (*π*). Nucleotide diversity represents the cumulative degree of genetic variation during evolution, while haplotype diversity reflects the probability that two randomly sampled alleles are different and is more relevant to population dynamics at short time scales (Leitwein et al., 2020; Pauls et al., 2013). Patterns of genetic diversity can be assigned to four categories (Grant & Bowen, 1998): (1) low *h* and low *π*, (2) high *h* and low *π*, (3) low *h* and high *π*, and (4) high *h* and high *π*. The results for all 39 *Gynaephora* populations as a whole were consistent with the fourth category, i.e., high *h* (>0.5) and high *π* (>0.5%), suggesting that QTP *Gynaephora* species was a large stable population with a long evolutionary history or secondary contact between differentiated lineages.

Among the four clades (A–D in Figures 1, 2, 3), Clade A showed the highest haplotype and nucleotide diversities, consistent with the hypothesis that source populations generally have high genetic diversity. Given that Clade A consisted of three morphologically described species, we proposed that the high level of genetic diversity found in Clade A may be related to secondary contact between these differentiated species/populations during glacial–interglacial cycle periods. Furthermore, it is likely that there are cryptic species within Clade A that were not resolved with the present mitochondrial data. Clade B (*G. alpherakii*) fell into the first category (low *h* and low *π*), suggesting a contribution of founder events in which new populations were established by a small number of individuals drawn from the large ancestral Clade A. In particular, only one exclusive haplotype was found in geographical populations AD, NQ, RW, SS, and XQ from Clade B, further confirming that genetic drift might eliminate many haplotypes, leaving only one haplotype in these populations. It is possible that Clade B, found in higher‐altitude areas, faced stronger environmental pressures, resulting in lineage‐specific haplotypes. Both Clades C and D were consistent with the second category, i.e., high haplotype diversity (*h* > 0.5) and low nucleotide diversity (*π* < 0.5%). This might be attributed to population expansion after population bottleneck followed by rapid population growth and the accumulation of new mutations (Grant & Bowen, 1998). High haplotype diversity was likely related to the high mitochondrial DNA mutation rate, as indicated by a haplotype network analysis (Figure 2) with 1–2 mutation steps before the generation of a new haplotype.

### 4.3 Isolation and subsequent divergence is the key speciation mechanism of the QTP *Gynaephora* species

The divergence between QTP *Gynaephora* species and non‐QTP species was estimated to occur in the early Pliocene (around 4.65 Ma), which was slightly younger than our previous estimate (Yuan et al., 2015). The genus *Gynaephora* likely originated in central Europe, as indicated by the biogeographic analysis (Figure 5). Therefore, ancestors of QTP *Gynaephora* arrived at the QTP during a period of uplift. The QTP has experienced uplifts many times; an intensive uplift probably began during the Miocene and the most intensive uplifts during the Pliocene and Pleistocene are known as the Qingzang Movement (3.6–1.7 Ma) and the Kun‐Huang Movement (1.2–0.6 Ma) (Shi et al., 1998; Zheng et al., 2000; Zhisheng et al., 2001). These intensive uplifts of the QTP were likely causes of interspecific divergence in QTP *Gynaephora* date to the early Pleistocene to Pliocene (2.08–0.53 Ma). In addition, changes in vegetation from forests to grasslands due to climate change during the Pleistocene (Wu et al., 2001) not only provided abundant food resources, an important basis for population expansion, but also likely provided new habitats, contributing to population differentiation and speciation. Therefore, we proposed that grassland caterpillars arrived at the QTP after the QTP uplifts and then gradually diverged, resulting in speciation influenced by plateau uplifts and associated climatic fluctuations.

Biogeographic analysis, divergence time estimation, and gene flow analysis showed that the region where *Gynaephora* first arrived was the interior of the QTP (the Sanjiangyuan region, Clade A in Figure 1) after its migration from Eurasia. This was also supported by the higher genetic diversity in Clade A than in the three other clades. Given the unidirectional gene flow from Clade A to Clade B, the QTP *Gynaephora* species may have remained in the interior of the QTP during the glacial period, rather than dispersing to low‐altitude environments. Although it is not clear whether the QTP formed a large ice sheet during the glacial period, some animals did remain in the QTP interior during these periods. Therefore, the Sanjiangyuan region was likely a refuge for QTP *Gynaephora* during glacial periods of the Pleistocene. Generally, postglacial differentiation could induce a decrease in genetic diversity along the expansion route, with an increase in the distance from refugia (Comes & Kadereit, 1998; Provan & Bennett, 2008). This hypothesis can explain the decrease in genetic diversity from Clade A to Clade B; however, it was difficult to explain the increasing trend from Clade B to Clade C. Both Clades C and D inhabited relatively low altitudes and showed relatively high genetic diversity, likely due to a high level of bidirectional gene flow between these two clades during the interglacial period. Significant genetic differentiation among the four clades and unidirectional gene flow (Clade A to Clade B and then Clade C), together with the observed association between genetic and geographic distances, indicated that geographic isolation and subsequent divergence was the dominant mode of speciation in the QTP *Gynaephora*, as has been proposed in some animal taxa endemic to the QTP (Favre et al., 2015; Lei et al., 2014; Wen et al., 2014).

## 5 CONCLUSION

In this study, we investigated the phylogeography and speciation of QTP *Gynaephora* based on two mitochondrial genes by extensive sampling. We recovered four monophyletic clades, indicating that the number of QTP *Gynaephora* species may be overestimated. The taxonomic status of the eight QTP *Gynaephora* species described based on morphological characteristics needs to be further studied by using a combination of morphological and nuclear data. High levels of genetic diversity detected in QTP *Gynaephora* may explain the high‐altitude adaptation and outbreaks in alpine meadows of the QTP. The ancestor of the QTP arrived in the region during the Pliocene, when the QTP had been uplifted, and gradually diverged, influenced by intensive plateau uplifts and associated climate fluctuations during the Pliocene and Pleistocene periods. Accordingly, isolation and subsequent divergence may explain speciation in QTP *Gynaephora*. This study is the largest phylogeographic analysis of QTP *Gynaephora* species to date and provides insights that may contribute to the control of these pests in alpine meadows.

## AUTHOR CONTRIBUTIONS

**Ming‐Long Yuan:** Conceptualization (lead); formal analysis (equal); funding acquisition (lead); methodology (lead); software (equal); supervision (lead); writing – original draft (lead); writing – review and editing (lead). **Ming‐Hui Bao:** Investigation (equal); methodology (equal); software (equal); visualization (equal); writing – original draft (equal). **Qi‐Lin Zhang:** Formal analysis (equal); investigation (equal); methodology (equal); visualization (equal); writing – review and editing (equal). **Zhong‐Long Guo:** Formal analysis (equal); investigation (equal); methodology (equal); visualization (equal); writing – review and editing (equal). **Min Li:** Data curation (equal); formal analysis (equal); investigation (equal); methodology (equal); visualization (equal). **Juan Wang:** Data curation (equal); formal analysis (equal); investigation (equal); methodology (equal); visualization (equal).

## CONFLICT OF INTEREST STATEMENT

The authors declare that they have no competing interests.

## Supporting information


Figure S1.

Figure S2.

Table S1.

Table S2.

Table S3.

## Data Availability

All sequences newly obtained in this study have been deposited in GenBank under accession numbers OP574353–OP574695 and OP577499–OP577841.
