# Supplementary figures and images for: Mitochondrial phylogeography of grassland caterpillars (Lepidoptera: Lymantriinae: Gynaephora) endemic to the Qinghai–Tibetan plateau
Source: Ecol Evol. 2024 Sep 15;14(9):e70270. doi: 10.1002/ece3.70270 (PMC11402507; doi:10.1002/ece3.70270)

all populations

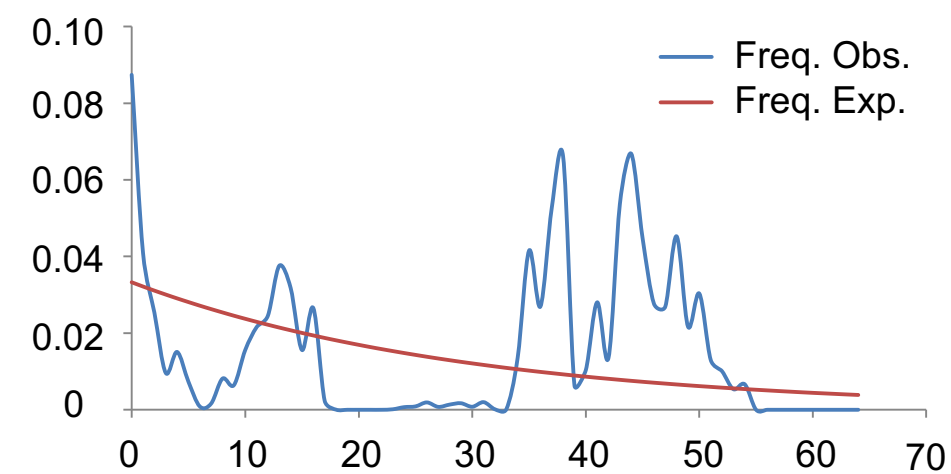

Clade C

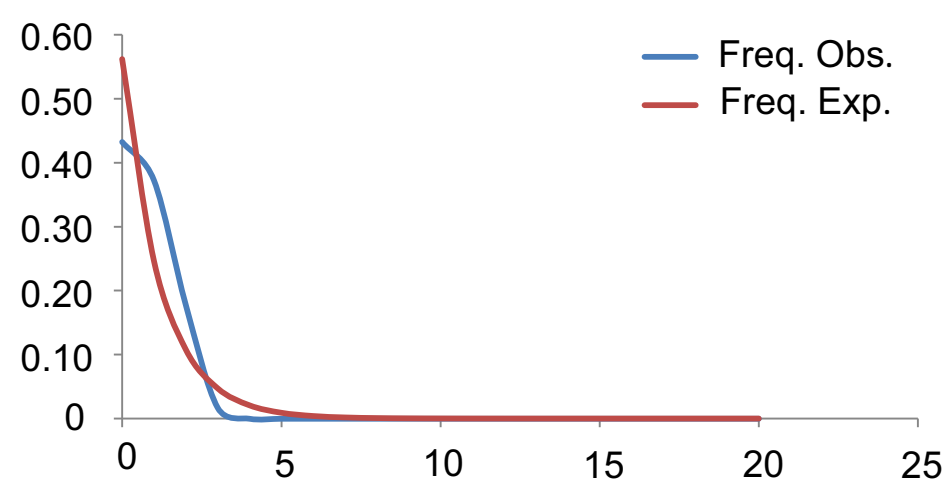

Clade A

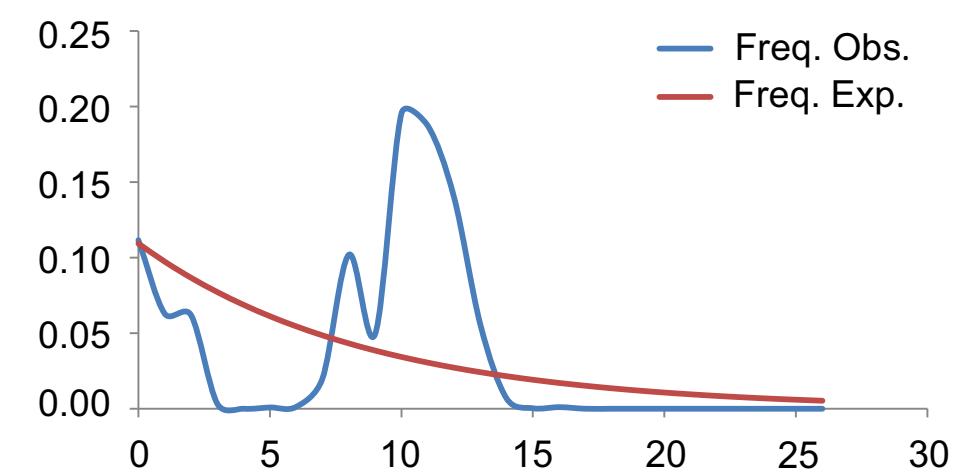

Clade D

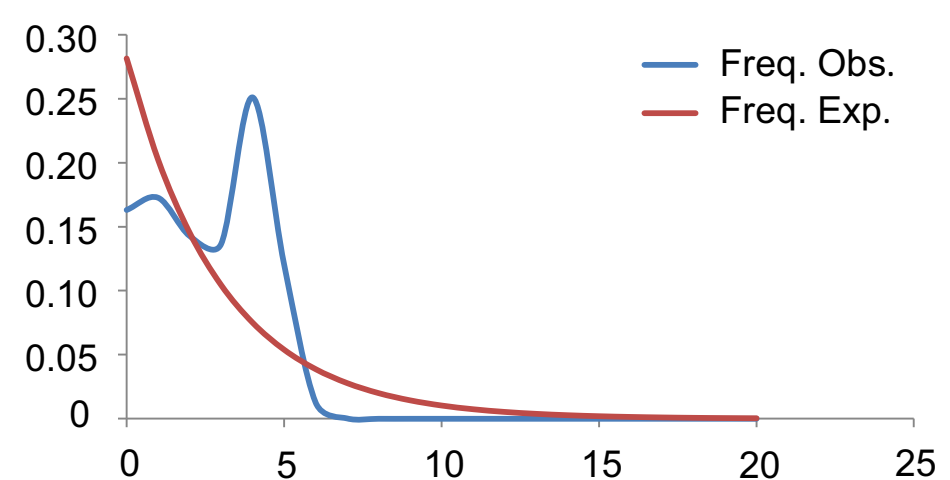

Clade B

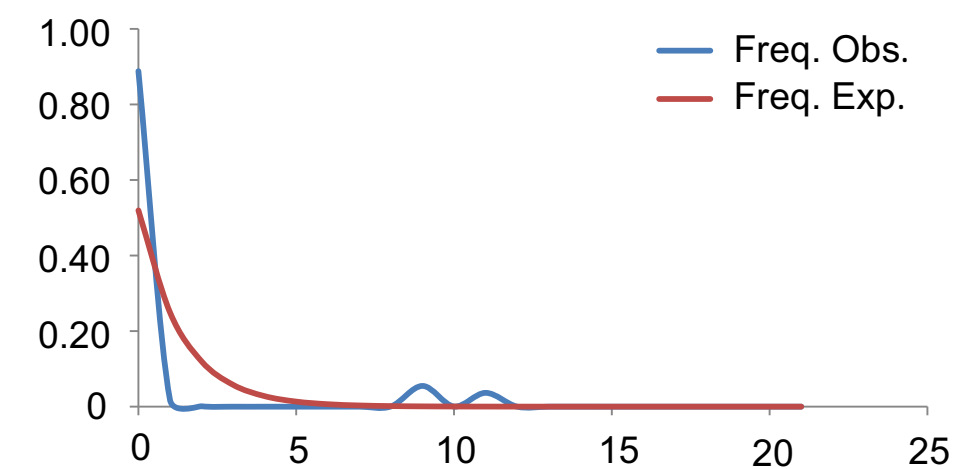

Supplement: Supplementary file 1 — Figure S1. [file ECE3-14-e70270-s005.pdf]
