# Supplementary material for: Mitochondrial phylogeography of grassland caterpillars (Lepidoptera: Lymantriinae: Gynaephora) endemic to the Qinghai–Tibetan plateau
Source: Ecol Evol. 2024 Sep 15;14(9):e70270. doi: 10.1002/ece3.70270 (PMC11402507; doi:10.1002/ece3.70270)

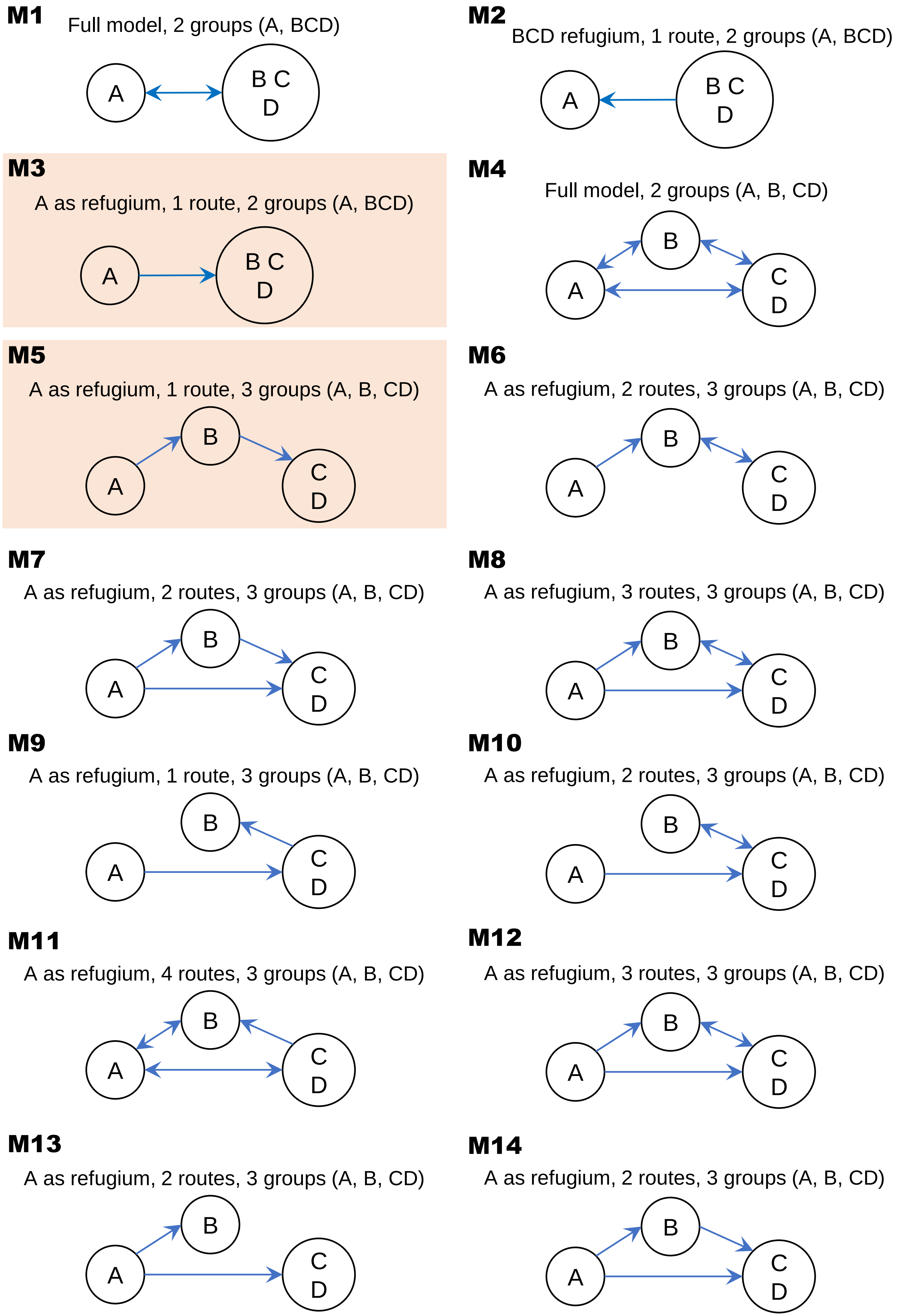
M1
Full model, 2 groups (A, BCD)
A
B C
D
M2
BCD refugium, 1 route, 2 groups (A, BCD)
A
B C
D
M3
A as refugium, 1 route, 2 groups (A, BCD)
A
B C
D
M4
Full model, 2 groups (A, B, CD)
B
A
C
D
M5
A as refugium, 1 route, 3 groups (A, B, CD)
B
A
C
D
M6
A as refugium, 2 routes, 3 groups (A, B, CD)
B
A
C
D
M7
A as refugium, 2 routes, 3 groups (A, B, CD)
B
A
C
D
M8
A as refugium, 3 routes, 3 groups (A, B, CD)
B
A
C
D
M9
A as refugium, 1 route, 3 groups (A, B, CD)
B
A
C
D
M10
A as refugium, 2 routes, 3 groups (A, B, CD)
B
A
C
D
M11
A as refugium, 4 routes, 3 groups (A, B, CD)
B
A
C
D
M12
A as refugium, 3 routes, 3 groups (A, B, CD)
B
A
C
D
M13
A as refugium, 2 routes, 3 groups (A, B, CD)
B
A
C
D
M14
A as refugium, 2 routes, 3 groups (A, B, CD)
B
A
C
D

M15

A as refugium, 2 routes, 3 groups (A, B, CD)

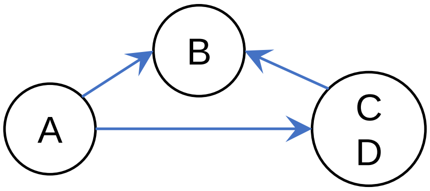

M16

A as refugium, 3 routes, 3 groups (A, B, CD)

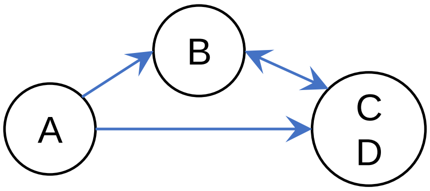

M17

A as refugium, 2 routes, 3 groups (A, B, CD)

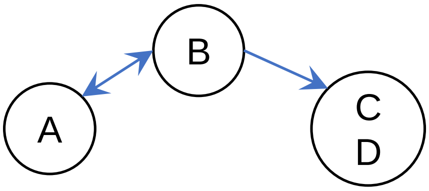

M18

Full model, 4 groups (A, B, C, D)

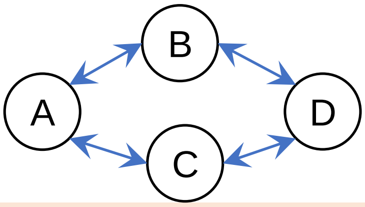

M19

A as refugium, 1 route, 4 groups (A, B, C, D)

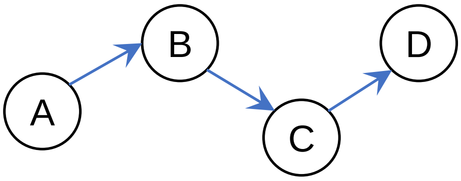

M20

A as refugium, 2 routes, 4 groups (A, B, C, D)

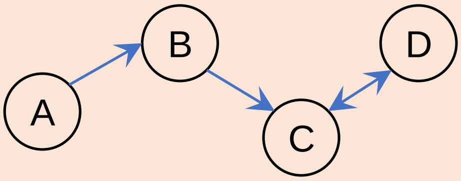

M21

A as refugium, 1 route, 4 groups (A, B, C, D)

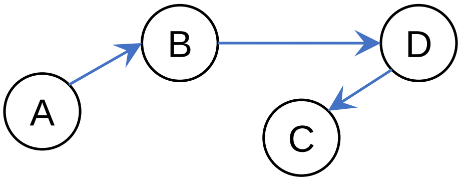

M22

A as refugium, 2 routes, 4 groups (A, B, C, D)

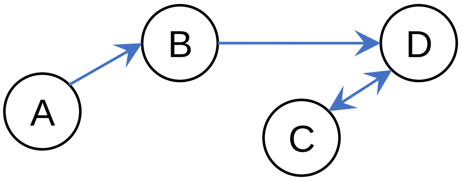

M23

A as refugium, 2 route, 4 groups (A, B, C, D)

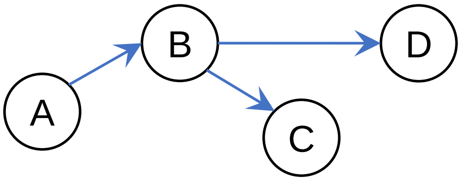

M24

A as refugium, 3 routes, 4 groups (A, B, C, D)

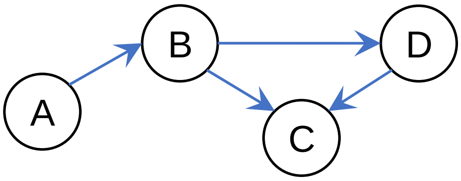

M25

A as refugium, 2 routes, 4 groups (A, B, C, D)

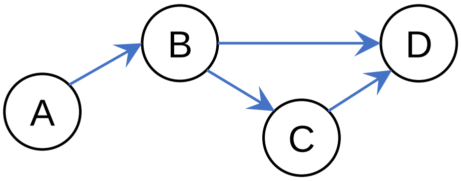

M26

A as refugium, 3 routes, 4 groups (A, B, C, D)

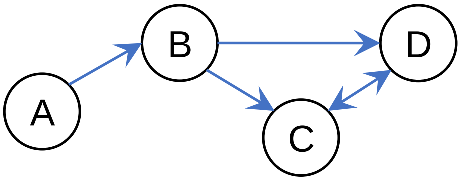

Supplement: Supplementary file 2 — Figure S2. [file ECE3-14-e70270-s001.pdf]
